# Self-leadership as an attribute of service leadership: Its relationship to well-being among university students in Hong Kong

**DOI:** 10.3389/fpsyg.2023.1088154

**Published:** 2023-01-20

**Authors:** Daniel T. L. Shek, Xiaoqin Zhu, Diya Dou, Lindan Tan

**Affiliations:** Department of Applied Social Sciences, The Hong Kong Polytechnic University, Kowloon, Hong Kong SAR, China

**Keywords:** life satisfaction, service leadership, positive youth development, pretest-posttest, leadership attitude, course evaluation

## Abstract

**Introduction:**

In the scientific literature, although conceptual models and empirical evidence have shown that leadership attributes are intimately linked to the well-being of followers, there is a lack of studies focusing on leadership in the service economy. According to the Service Leadership Theory, service leadership is a process that satisfies the needs of self, others, and systems (teams, organizations, communities, and societies) in ethical ways that is characterized by leadership competence, character, and care. With specific reference to self-leadership emphasized in service leadership, higher levels of service leadership attributes should promote personal well-being. However, the relationships between “service leadership attributes” and “well-being” in leaders at the intrapersonal level in leadership education among Chinese university students are rarely examined.

**Methods:**

In this study, we collected data from 198 students to understand the linkages between “service leadership attributes” and “well-being” in university students taking a course on service leadership. For tracking changes in students, we collected both pretest and posttest data on validated measures of “service leadership attributes” (i.e., “knowledge,” “attitude,” and “behavior”) and “well-being” (i.e., “positive youth development attributes” and “life satisfaction”).

**Results:**

Results showed that the posttest scores on all three domains of “service leadership attributes” as well as two dimensions of “well-being” encompassing life satisfaction and positive youth development attributes were higher than the respective pretest scores, suggesting that students experienced a shift in a positive direction after taking the course. Cross-lagged analyses showed that pretest service leadership attitude and behavior predicted posttest positive youth development attributes; pretest service leadership behavior predicted posttest life satisfaction. Pretest life satisfaction also predicted posttest service leadership behavior.

**Discussion:**

Findings suggest that there is an intimate relationship between “service leadership attributes” and “well-being” in the “pre-work” context among university students.

## 1. Introduction

Leaders undeniably perform a crucial role within an organization. In addition to setting organizational goals and strategies, leaders also consciously shape the development of their followers through supervision, coaching, training, and role modeling. Besides, leaders influence followers through their leadership attributes. For example, leaders focusing on achievements with authoritarian control alone would create much stress for the followers. On the other hand, leaders focusing on achievement with care about the followers would create support, cohesion, and positive energies among the followers. Hence, it is important to understand the relationship between leadership attributes and mental well-being in the field of leadership.

### 1.1. Leadership style, self-leadership, and well-being

In the scientific leadership literature, studies have shown the association between the attributes of leaders and the development of followers and coworkers. Montano et al. (2017) conducted a meta-analysis to understand the linkages between leadership attributes (transformational, relationship-oriented, task-oriented, destructive, and leader-member exchange styles) and job performance as well as mental health of the followers (indexed by emotional problems, burnout, stress, wellness, mental functioning, and health problems). They found that while there were positive relationships between some leadership attributes (e.g., transformational and relations-oriented leadership) and mental health, a negative correlation was detected between destructive leadership attributes and mental health. They also found that mental health acted as a significant mediator in the association between leadership attributes and the job performance of subordinates. After reviewing studies and theories in the leadership field, Inceoglu et al. (2018) summarized that different leadership attributes (change-oriented, task-oriented, relational-oriented, passive leadership, and others) influenced different aspects of the well-being of followers, including hedonic, eudaimonic, negative, and physical indicators. They also identified several mediators in this relationship, including social-cognitive, affective components, motivating features, identification elements, and relational factors. They remarked that “as a starting point for future research, leadership researchers will need to take employee well-being more seriously as a criterion in and of itself—as an end goal rather than merely as a means to higher performance” (Inceoglu et al., 2018, p. 189).

Additionally, leadership qualities not only correlate with the well-being of subordinates, but also shape leaders’ own well-being. This association demonstrates the importance of self-leadership in promoting personal development. Manz (1986) defined self-leadership as “a comprehensive self-influence perspective that concerns leading oneself toward performance of naturally motivating tasks as well as managing oneself to do work that must be done but is not naturally motivating” (p. 589). There are two key categories of strategies underlying self-leadership, including “behavior-focused strategies” (e.g., “goal setting,” “rehearsal,” “self-reward,” and “self-punishment”) and “cognitive-focused strategies” such as natural rewards (Manz and Sims, 1991, p. 24). Krampitz et al. (2021) argued that “effective leadership starts with effective self-leadership” (p. 22). In a meta-analysis involving 11 studies, Krampitz et al. (2021) showed that leadership interventions were effective in developing self-leadership skills. As concluded by Dolbier et al. (2001), “self-leadership can be learned and applied, coupled with the findings relating self-leadership to positive psychological, health, and work outcomes, the practical application of self-leadership is a worthwhile avenue to pursue” (p. 483). Obviously, service leadership education is a good way to promote self-leadership in leaders, which would eventually promote psychological well-being.

In the fields of management and business, there is support for the positive effects of self-leadership on individual and team performance, organizational sustainability, and national culture (Goldsby et al., 2021) as well as well-being and mental health (Uzman and Maya, 2019; Krampitz et al., 2021). Research shows that self-leadership has positive impacts on the lives of individuals. Focusing on adolescents, Uzman and Maya (2019) revealed a positive relationship between self-leadership strategies and well-being indexed by life satisfaction and self-esteem in university students. Similarly, Dolbier et al. (2001) demonstrated that self-leadership positively correlated with college students’ hardiness, dispositional optimism, and health conditions but was negatively related to interpersonal mistrust, illness symptoms, and perceived stress.

The beneficial connection between self-leadership and well-being was also reported in studies involving adults. Dolbier et al. (2001) reported that self-leadership correlated positively with working relationships, job satisfaction, and perceived wellness. In another study in Finland, Sjöblom et al. (2022) showed that self-leadership strategies were negatively associated with burnout but positively associated with perceived meaningfulness of work. In addition, studies also documented the availability of self-leadership as the protective factor for psychological health: Şahin and Gülşen (2022) reported that self-leadership mediated the influence of basic psychological needs satisfaction and occupational adaptability. Based on a sample of German employees, Seubert et al. (2017) showed that “cognition-based strategies” significantly buffered the impact of overload at work on exhaustion tendencies. However, Roberts and Foti (1998) examined the moderating role of self-leadership between job structure and job satisfaction in 76 employees and surprisingly found that “high self-leaders who worked in highly structured work environments reported the least amount of satisfaction with their jobs” (p. 263).

### 1.2. Understanding the linkage between service leadership and well-being via the self-leadership perspective

Notably, from the review above, we can tentatively conclude that although there are intimate relationships between leadership style and the well-being of the followers, as well as the benefits of self-leadership on leaders’ well-being, there are several gaps in the literature. First, although there are theoretical and empirical works on the linkages among diverse leadership attributes and follower well-being based on some leadership models (e.g., transformational leadership and servant leadership), not much work has been done based on leadership grounded in the service economy. With the rapid growth of the service economy, how leadership attributes in the context of the service economy (i.e., “service leadership attributes”) are related to the well-being of followers is an important question to be addressed.

In response to the rapid development of service economies worldwide, Dr. Po Chung, Co-founder of DHL International Limited and Chairman Emeritus of DHL Express (Hong Kong) Limited, proposed the “Service Leadership Theory” (Shek et al., 2018a, 2022a). In this theory, it is asserted that service leadership is a process that satisfies the needs of self, others, and systems (teams, organizations, communities, and societies) in ethical ways (Shek et al., 2018a). It also highlights seven core beliefs (e.g., “everyone can be a leader,” “a service leader is a service provider,” and “leadership includes self-serving efforts for continuous improvement”) and maintains that an effective service leader has three attributes (“competence,” “character,” and “care,” 3 Cs) determining the leader-follower relationship and related outcomes.

Leadership competencies generally refer to generic competencies that enable leadership effectiveness, including management skills, problem-solving capabilities, and skills that would facilitate the production process. In particular, Shek et al. (2015b, 2022a) focus on the prominence and valuation of “soft skills” or “21st Century skills,” including “adversity quotient” (AQ), “emotional quotient” (EQ), and “spiritual quotient” (SQ). Character refers to moral character attributes such as integrity, a sense of righteousness, and courage. These character attributes can be understood through both Western (e.g., character strengths under the “Values in Action,” Peterson and Seligman, 2004) and Chinese frameworks (e.g., traditional Chinese virtues, Shek et al., 2015c). Finally, an effective service leader exercises care about different stakeholders, particularly concern about the followers. Shek et al. (2021) further studied the origin of the “Service Leadership Theory.” They concluded that the model is humanistic in nature with a strong emphasis on the importance of character and values.

Although other propositions emphasize distributed leadership (i.e., every employee takes leadership responsibilities) and service orientation in the service era (e.g., Grönfeldt and Strother, 2006; Snell et al., 2015), it can be argued that the “Service Leadership Theory” is unique in integrating all essential propositions regarding the requirements of the growing service economies. Shek et al. (2015a) compared the “Service Leadership Model” with some other contemporary leadership models, including the “trait approach,” “servant leadership,” “spiritual leadership,” “authentic leadership,” “ethical leadership,” “transformational leadership,” “charismatic leadership,” and “top-down leadership approach.” “Service Leadership Theory” emphasizes that leadership can be learned and is not based on inherent traits or charisma. Besides, this theory involves a comprehensive understanding of leadership qualities, including competencies, character, and a caring disposition, in contrast to other theories that focus on specific aspects of leadership, such as spiritual leadership and ethical leadership. The Service Leadership Theory also defines leadership at the individual, interpersonal, and systemic levels, unlike theories that only consider leaders’ personal characteristics and leader-follower relationships, such as authentic leadership. Additionally, service leadership emphasizes the importance of self-leadership and ethical self-care, while other theories, such as transformational leadership and top-down leadership, do not specifically take this perspective into account. To conclude, “Service Leadership Theory” is unique in terms of incorporating people orientation, service orientation, system orientation, focus on the three “Cs” of leadership (competencies, character, and care) and personal qualities of leaders, the belief that “everyone can be a leader,” emphasis on the significance of self-leadership, urge for constant self-improvement, emphasis on the significance of mentoring followers, and innovative integration of traditional Chinese virtues (Shek et al., 2015c). As such, it is interesting to ask how the “service leadership attributes” are related to the well-being of the leaders as well as followers.

Second, the linkage between leadership and well-being has been primarily addressed within the interpersonal context involving leaders and followers (or coworkers). For example, based on meta-analysis of 209 studies of Li P. et al. (2021) across different cultures, positive leadership styles (e.g., ethical and servant leadership) are universally positively associated with subordinates’ work engagement and well-being. While this is a legitimate focus, we should ask another question—are leadership attributes related to the well-being of the leaders themselves (i.e., intrapersonal context)? According to the self-leadership perspective, before leading others, one has to learn how to lead oneself. Hence, the connection between leadership attributes and well-being in terms of the intrapersonal perspective should be examined. However, few researchers have examined the linkages between diverse leadership qualities (e.g., resilience) and well-being (e.g., life satisfaction). In a meta-analysis, Hoch et al. (2018) examined whether authentic leadership, ethical leadership, servant leadership, and transformational leadership are differently associated with organizational outcomes. Results supported the distinctiveness of servant leadership in explaining the linkage between leadership qualities and a wide range of organizational outcomes. As such, by examining the association between service leadership (in which character and care are two dominant dimensions) and personal well-being, the current study will contribute to the discussion on the role of positive leadership in the promotion of well-being in the leaders themselves.

Third, most of the studies on leadership and worker well-being were conducted in Western contexts with very few explorations rooted in Chinese settings. In their meta-analysis, Li P. et al. (2021) highlighted the importance of cultural characteristics in shaping the associations between leadership and organizational well-being. There are several reasons why this issue should be examined in Chinese people. Firstly, while conventional leadership theories often adopt individualistic assumptions, Chinese culture is more collectivist in nature (Shek et al., 2022c). Besides, given the huge population size of Chinese people, there is a need to examine whether the relationships between leadership and well-being apply to the Chinese context. Secondly, as China is experiencing a booming growth shift from the manufacturing economy to the service economy, understanding how service leadership contributes to leadership well-being is a timely question to be answered. Thirdly, the focus on character and caring dispositions are indeed consistent with the conception of leadership under Confucian thoughts. Finally, as there remain few studies on leadership psychological attributes in the Chinese setting, we need more effort to build up the database in this area.

The fourth limitation of the literature is that few studies have examined this issue in the “pre-work” context. According to the “Service Leadership Theory,” everybody is (and can be) a leader. Hence, university students are (and can be) leaders. According to the self-leadership view that everyone is a leader of himself/herself, university students can be regarded as leaders at least leading themselves. Based on these beliefs, it is legitimate to ask whether service leadership education has any impact on the well-being of university students. Theoretically, examination of this issue in the higher education context would expand our understanding of the leadership-well-being relationship beyond the “work” context to the “pre-work” context. Practically, research findings would provide insight into the role of leadership education in nurturing student well-being in the higher education sector.

Finally, under the turbulence of the pandemic when courses are moved online, we rarely understand the role of leadership education in well-being and how “service leadership attributes” are related to individual “well-being” when the online mode is adopted. In a study evaluating the outcomes of synchronous and asynchronous online teaching modes during the pandemic, Zhu et al. (2021) revealed that the two teaching modes showed positive impacts. Using pretest and posttest data, participants exhibited significant improvement either in their well-being or their “service leadership attributes.” Using the client satisfaction approach, students also responded positively with regard to course design, instructor performance, and personal gains obtained from the course. In another recent study examining the effectiveness of a leadership course, Chai et al. (2022) similarly revealed that 630 students showed improvement in life satisfaction, positive youth development traits, and desirable graduation qualities as a result of completing the course. Besides, students also demonstrated high satisfaction with curriculum design, instructor performance and overall payoff derived from the course. There are also some studies showing the effectiveness of service-learning courses on service leadership (Lin et al., 2022; Zhu et al., 2022; Shek et al., 2022b).

### 1.3. “Service leadership attributes” and “well-being”

With reference to the “Service Leadership Model” (Chung and Bell, 2012; Shek et al., 2015a), there are three basic qualities of accomplished service leaders – “competence,” “character,” and “care.” Obviously, we can ask whether there are any relationships between these “service leadership attributes” and “well-being,” particularly in university students.

Concerning the relationship between resilience and well-being in young people, there are research findings demonstrating that these two domains are positively related in Nigerian students (Ifeagwazi et al., 2015) and Australian students (Turner et al., 2017). Similar findings were observed in adults. Zhao et al. (2021) reported that while four dimensions of resilience (persistence of effort, strength, tenacity, and optimism) were positively associated with life satisfaction among Chinese adults, only tenacity and optimism significantly predicted life satisfaction. Resilience also plays a protective role in well-being in leadership settings. Zhou et al. (2022) revealed that resilience was associated positively with caring ability and self-learning in student leaders. Hudgins (2016) revealed that resilience in nursing leaders was positively correlated with their job satisfaction. Walumbwa et al. (2010) also found that police officers’ resilience predicted followers’ job performance and psychological capital (e.g., efficacy, hope, and optimism). Finally, De Clercq et al. (2021) demonstrated that employee resilience in the telecommunications and banking sectors mitigated the negative effect of perceived leader arrogance on employee distrust for leaders’ consistency in action.

There is also evidence of a strong positive association between emotional competence and well-being in managers (Sharma, 2011). Kafetsios et al. (2011) also found that supervisors’ emotional use capacity was positively negatively correlated with subordinates’ personal fulfillment and job satisfaction, while being associated negatively with followers’ depersonalization in the school context. In a meta-analysis, Miao et al. (2016) concluded that leaders’ emotional intelligence strongly predicted followers’ job satisfaction, and followers’ emotional intelligence played a significant mediating role in this link. Andrei et al. (2022) similarly revealed that trait emotional intelligence moderated the direct predictive effects of unemployment on the quality of life during the pandemic.

Tracking 116 general adults living in Europe, Berking et al. (2014) showed that deficits in emotion regulation skills were strongly associated with depressive symptoms, with incompetent emotion regulation predicting the severity of depression after 5 years. Llinares-Insa et al. (2020) also reported that emotional intelligence was positively correlated with subjective well-being in parents and parental moods played an important mediating role in the influence of emotional intelligence on subjective well-being. Recently, survey of Chinese university students of Li N. et al. (2021) showed that self-perceived emotional competence (emotion appraisal, regulation, and use) mediated the association between crisis exposure during COVID-19 and anxiety as well as depressive symptoms. However, it is noteworthy that some studies showed the possible negative effects of emotion induction (e.g., Petrides and Furnham, 2003).

Finally, studies have shown that spirituality, such as the meaning of life, was related to well-being in students (Shek and Lin, 2015; Pant and Srivastava, 2019). For example, Kleiman and Beaver (2013) showed that the presence of life meaning as a “suicide resiliency factor” significantly predicted a decrease in suicidal ideation over time. Similar findings were reported based on adults (Schnell and Krampe, 2020; Moafi et al., 2021). In the leadership literature, many scholars highlighted the benefits of spirituality for well-being in leadership contexts. As Fry (2003) asserted, “spiritual leadership comprises the values, attitudes, and behaviors necessary to intrinsically motivate oneself and satisfy fundamental needs for spiritual well-being, through calling and membership” (p. 711). Based on civil servants in Indonesia, Wahyono et al. (2021) reported that spiritual leadership promoted workplace spirituality and job satisfaction as well as reduced deviant behavior in the workplace. However, there are findings suggesting that while leadership spirituality promotes work meaning, it may also create negative emotions, particularly when leaders abuse their power (Rego et al., 2008; Chaston and Lips-Wiersma, 2015).

Regarding character, studies have examined the linkage between character and well-being in light of the character strengths framework (Peterson and Seligman, 2004). In the non-clinical populations, positive relationships between a wide range of character strengths and well-being were reported both in adults (Martínez-Martí et al., 2020) and young people (Shoshani and Slone, 2013; Zhang and Chen, 2018). In the clinical populations, there is also support for the beneficial effect of character strengths on well-being in first-episode psychosis patients (Browne et al., 2018). Umucu et al. (2021) also showed that higher levels of comprehensive character strengths could buffer the adverse impairment of COVID-19 stress on patient well-being, including positive emotions, engagement, and relationships. The beneficial role of character on well-being has also been reported in the leadership literature, particularly in relation to ethical leadership. Generally speaking, ethical leadership attributes had a positive association with employee job satisfaction and other well-being components in Pakistan (Aftab et al., 2022), Turkey (Demirtas et al., 2017), and the United States (Sosik et al., 2019).

In a cross-cultural context, character is not only identified as providing a “compass of a good life” (Martínez-Martí et al., 2020; Littman-Ovadia et al., 2021) in the Western contextual findings, but also noted for its crucial value as a “compass of moral” (Shek et al., 2022a) in the Chinese traditional culture. Advocating the presence of the philosophy of Confucianism in Chinese culture as the ideal of how the Chinese deal with themselves, others, and the world, Shek et al. (2013) pioneered a discourse on the relationship between adolescent mental health and the key Confucian character strengths. These included “zhong (loyalty), xiao (filial piety), ren (benevolence), ai (affection), xin (trustworthiness), he (harmony), ping (peace), li (propriety), yi (righteousness), lian (integrity), chi (shame), zhi (wisdom)” (p. 336).

Empirically, there are studies demonstrating that filial piety (Yang and Wen, 2021), interpersonal harmony (Hsiao et al., 2006), and forgiveness (Chan, 2013) were positively associated with well-being. To conclude, Zhou et al. (2021) revealed that moral character attributes, including traditional Chinese virtues, which embodied as polite, loyal, forgiving, sense of justice among others, not only positively associated with life satisfaction, but also significantly predicted life satisfaction among Hong Kong students (*N* = 2,474), and adolescent responsible behavior could significantly mediate such a link.

Finally, caring attributes include love, listening, and empathy (Shek et al., 2022a), the key properties of Positive Youth Development (PYD). Caring attributes have been found to contribute to the well-being of the individual, family, and community. Research showed that love (Culshaw and Kurian, 2021), caring climate (Fry et al., 2012), family support displayed as active listening (Alsawy et al., 2020), and community support promoted community well-being (Berardi et al., 2020).

In the leadership literature, research also suggests that leadership care was significantly correlated to staff well-being (Kock et al., 2019). In Germany, Jacobs et al. (2013) revealed that a higher level of transformational leadership, characterized by caring and considering subordinates’ feelings, in particular, was significantly and positively correlated with higher employee psychological well-being. In Denmark, Munir et al. (2010) also showed a negative link between transformational leadership style and employee depression over time. Moreover, Kock et al. (2019) revealed not only a positive association between empathetic leadership and follower job satisfaction, but also a significant moderating effect of empathetic leadership in the link between employee job satisfaction and innovative behavior. Nevertheless, there are views suggesting that over-use of care, such as “compassion fatigue” and “overloaded supervisor care,” may lead to negative outcomes (e.g., Duarte and Pinto-Gouveia, 2017). For example, Boekhorst et al. (2021) showed that while managerial caring behavior was positively correlated with employee vitality in the workplace, its negative effect appears to be more pronounced when there were employee-rated manager overload and employee guilt.

### 1.4. The present study

To understand the relationship between “service leadership attributes” and “well-being” based on university students during the pandemic, we asked two research questions:

Research Question 1: Did students who took the “Service Leadership” course *via* online and hybrid modes during the pandemic demonstrate an improvement in their “service leadership attributes” as well as “well-being”? Based on previous studies and predictions of the “Service Leadership Theory,” we anticipated that students would exhibit improvements in “service leadership attributes” (Hypothesis 1a) and “well-being” (Hypothesis 1b) after participating in the course.

Research Question 2: What are the inter-relationships between “service leadership attributes” and “well-being”? According to “Service Leadership Theory” and previous studies, we predicted the existence of positive correlations between “service leadership attributes” and “well-being” at the pretest and posttest (Hypothesis 2a). Based on the work of Zhu and Shek (2021b), we also expected that service leadership attitudes and behavior at the pretest would predict well-being at the posttest (Hypothesis 2b).

By answering the above two questions, the present study contributes to the existing literature by delineating whether university students demonstrate any changes regarding service leadership qualities and well-being after taking a leadership course during the pandemic. Answers to the second research question also shed light on the cross-sectional and longitudinal associations between service leadership attributes and well-being in the “pre-work” context among Chinese university students at the intrapersonal level from a self-leadership perspective.

## 2. Methods

To understand the linkages between “service leadership attributes” and “well-being,” we utilized data collected from university students who enrolled in the “Service Leadership” offered in the summer term of 2021 and 2022. “Service Leadership” is a three-credit General Education course introducing the basics of “Service Leadership.” Over 2 years, a total of 198 students took the course. For tracking changes in students, we collected both pretest and posttest data on validated measures of “service leadership attributes” (i.e., “knowledge,” “attitude,” and “behavior”) and “well-being” (i.e., “positive youth development attributes” and “life satisfaction”). Using pretest and posttest data, we also examined the relationship between “service leadership attributes” in the three domains and “well-being” in two areas over time.

The “Service Leadership” course is a three-credit elective course that consists of 13 lectures of 3 h each, addressing a broad scope of service leadership-related knowledge. The lectures introduce the transition from manufacturing economies to service economy economies, seven essential beliefs (e.g., “everyone can be a leader”), key determinants of effective service leadership including competence (“intrapersonal competence” and “interpersonal skills”), character (different character strengths and traditional Chinese virtues), and care (“empathy” and “active listening”), and similarities and differences between “Service Leadership Theory” and other high-impact leadership theories (e.g., spiritual and servant leadership). Furthermore, real-life scenarios and examples are incorporated into different class activities (e.g., group discussions and case studies) to deepen students’ learning and help them apply the knowledge in daily life.

Notably, the course adopts a student-centered teaching pedagogy characterized by interactive teaching and learning methods, such as reflective learning (e.g., self-reflection on own leadership practices and service leadership qualities), learning collaboratively (e.g., drawing, projects, and presentations in the group format), critically thinking (e.g., encouraging critical evaluate of various leadership theories and attributes), and other in-class interactions (e.g., role play, debate, and games). Affected by the COVID-19 pandemic and the associated measures of social distancing, the “Service Leadership” course was offered to students through a hybrid mode blending physical and online teaching since the 2019–2020 summer semester with in-class interactive activities being refined to an online version (Zhu et al., 2021).

### 2.1. Participants and procedures

In two summer semesters in the academic years of 2020–2021 and 2021–2022 academic years, the “Service Leadership” course was provided through an online teaching format over a period of 7 weeks (i.e., two lectures were offered per week). For the purpose of assessing course effect in terms of changes in students after taking the course and investigating the association between “service leadership attributes” and “well-being” (i.e., the two aforementioned research questions), a one-group pretest-posttest design was employed in this study. Despite the intrinsic shortcomings of this type of pre-experimental design as a result of not involving control groups (Campbell and Stanley, 1996), it is quite feasible and practically preferred, especially in educational settings. Particularly, Thyer (2002) pointed out several myths in evaluating changes in psychosocial outcomes, such as “you must control for the most relevant threats to internal validity” or “you must randomly assign clients to various control and experimental groups” (pp. 12–13). Of course, he also acknowledged the limitations of the one-group pretest-posttest design and the meaningful usage of evaluation designs without a control group. As such, scholars devoted to educational and other social science fields like social work and youth services have advocated and adopted this research design in evaluating service or educational effectiveness (e.g., McElwain et al., 2016; Shek and Zhu, 2018; Zhu et al., 2022). In the present investigation, comparisons between the pretest and the posttest scores are expected to at least partially reflect students’ changes as a result of service leadership education, as other confounding effects (e.g., maturation) are less likely to fully operate in such a short period (i.e., 7 weeks).

In each summer semester, students enrolled in the course were invited to participate in an online pretest survey to be completed within a week prior to the first lecture and to finish the posttest within a week of completing the final lecture. Before responding to survey questions, students provided their consent after reading an online information sheet that explained key principles upheld in collecting, analyzing, and using the data, including voluntary participation, anonymity, and confidentiality. The “Institutional Review Board (and its Delegate)” at the authors’ university approved the present study.

In the summer semester of the 2020–2021 year, among the 118 enrolled students, 111 (response rate = 94.07%), and 100 (response rate = 84.75%) completed the pretest and posttest, respectively. A total of 93 cases (Mean age = 19.17 ± 1.51 years) were successfully matched (female = 55, 59.14%). In the summer semester of the 2021–2022 year, among the 113 enrolled students, 110 (response rate = 97.35%), and 107 (response rate = 94.69%) completed the pretest and posttest, respectively. A total of 105 cases (Mean age = 19.99 ± 1.64 years) were successfully matched (female = 44, 41.90%). The present study utilized the matched sample in two academic years (total N = 198, mean age = 19.61 ± 1.64 years, 50.00% female students).

### 2.2. Measures

The same indicators measuring the “service leadership attributes” (indexed by “knowledge,” “attitude,” and “behavior”) and “well-being” [indexed by “life satisfaction” as a measure of hedonic well-being and “positive youth development (PYD) attributes” as a measure of eudaimonic well-being] were employed in the pretest and posttest questionnaires.

*Service Leadership Knowledge (SLK)* was assessed using the “Service Leadership Knowledge Scale” consisting of a set of 40 multiple-choice questions on service leadership concepts and knowledge points (e.g., the underlying economic, social, and cultural context in which the service economy is rooted) mentioned in the lectures (Shek et al., 2018c). Students’ responses to each question were coded as “0″ or “1″ if the answer was incorrect or correct, respectively. The total score of SLK betwixt 0 and 40. This scale showed adequate psychometric properties in the validation study (Shek et al., 2018c) and had favorable reliability in previous studies (Zhu et al., 2021; Zhu and Shek, 2021b). As depicted in Table 1, the knowledge scale also had adequate internal consistency in both the pretest (α = 0.94; mean inter-item correlation = 0.30) and posttest (α = 0.95; mean inter-item correlation = 0.35) in the present study.

**Table 1:** Reliability of measures and participants’ changes from the pretest to posttest (*N* = 198).

Measures	Pretest		Posttest		*F*	*η*^2^_p_
	*M* (*SD*)	α (*M*_IIC_)	*M* (*SD*)	α (*M*_IIC_)		
Service leadership attributes					8.54^***, a^	0.12
SLK	27.68 (9.71)	0.94 (0.30)	29.10 (10.33)	0.95 (0.35)	5.99^*^	0.03
SLA	4.87 (10.52)	0.95 (0.55)	4.96 (0.65)	0.96 (0.59)	5.33^*^	0.03
SLB	4.67 (0.55)	0.95 (0.52)	4.86 (0.64)	0.97 (0.63)	20.17^***^	0.09
Positive youth development attributes					5.91^***, b^	0.11
CBC	4.58 (0.58)	0.90 (0.51)	4.79 (0.66)	0.93 (0.61)	16.65^***^	0.08
PI	4.40 (0.78)	0.86 (0.57)	4.64 (0.80)	0.89 (0.63)	6.13^***^	0.09
GPYD	4.54 (0.53)	0.87 (0.33)	4.65 (0.62)	0.88 (0.39)	7.35^**^	0.04
TPYD	4.53 (0.55)	0.94 (0.39)	4.69 (0.62)	0.96 (0.47)	15.50^***^	0.07
Life satisfaction	4.10 (0.88)	0.92 (0.69)	4.37 (0.92)	0.92 (0.70)	20.58^***^	0.10

SLK, service leadership knowledge; SLA, service leadership attitude; SLB, service leadership behavior; CBC, cognitive-behavioral competence; PI, positive identity; GPYD, general positive youth development qualities; TPYD, total positive youth development score; and M_IIC_, mean inter-item correlation; ^a^adjusted Bonferroni value (0.017), ^b^adjusted Bonferroni value (0.013); ^*^p < 0.05, ^**^p < 0.01, and ^***^p < 0.001.

*Service Leadership Attitude (SLA)* was gaged from the “Service Leadership Attitude Scale” containing 23 items on a six-point scale (“1” = “strongly disagree”; “6” = “strongly agree”). Sample items included “a good leader listens to his/her subordinates’ views” and “everyone has the potential to be a leader.” The scale has been validated in samples of Chinese university students based in Hong Kong (Shek and Chai, 2019) and demonstrated satisfactory psychometric characteristics in prior research (Zhu et al., 2021; Zhu and Shek, 2021b). For the present research, the scale’s Cronbach’s alpha values were above 0.95 on two assessment occasions (see Table 1), indicating the scale’s good internal consistency.

*Service Leadership Behavior (SLB)* was measured by a 19-item “Service Leadership Behavior Scale,” which has also been validated among Hong Kong Chinese university students (Shek et al., 2018b, 2019) and utilized to assess students’ service leadership behaviors covering the three Cs (competence, character, and care) in past studies (Zhu et al., 2021; Zhu and Shek, 2021b). Students were invited to rate each item (e.g., “I often try my best to help other people to overcome difficulties” and “I learn through reflecting on my experiences”) on a six-point scale (“1″ = “strongly disagree”; “6″ = “strongly agree”). This scale exhibited good reliability as indicated by Cronbach’s alpha values (0.95 and 0.97) and mean inter-item correlation (0.52 and 0.63; see Table 1) in this study.

*Life Satisfaction* was measured by the five-item Chinese “Satisfaction with Life Scale,” which assessed people’s cognitive ratings regarding their overall quality of life (Sun and Shek, 2010). Students rated each statement (e.g., “the conditions of my life are excellent”) using a six-point scale (“1” = “strongly disagree”; “6” = “strongly agree”). This instrument has been applied extensively in research involving Chinese young people and demonstrated adequate reliability (e.g., Zhang, 2016; Shek et al., 2017; Zhu and Shek, 2020). In this study, this scale also certainly exhibited good internal consistency at pretest (α = 0.92; mean inter-item correlation = 0.69) and posttest (α = 0.92; mean inter-item correlation = 0.70; see Table 1).

*PYD Attributes* were measured by a total of 10 subscales selected from the validated “Chinese Positive Youth Development Scale” (Shek and Ma, 2010), which followed previous practices in assessing PYD attributes in leadership education (Lin and Shek, 2019; Zhu and Shek, 2021b). The 31 items in the 10 subscales evaluated 10 corresponding PYD attributes, such as “emotional competence” and “resilience,” which can be further grouped under three “higher-order factors.” The first “higher-order factor” was “cognitive-behavioral competence” (CBC) summarizing “cognitive competence,” “behavioral competence,” and “self-determination.” The second “higher-order factor,” namely “positive identity” (PI), combined “clear and positive identity” and “belief in the future.” The third “higher-order factor” entitled “general PYD qualities” (GPYD) summed up five individual PYD attributes, including “emotional competence,” “social competence,” “moral competence,” “resilience,” and “spirituality (e.g., life meaning and purpose).” In the present study, we utilized a six-point scale (“1” = “strongly disagree”; “6” = “strongly agree”) to score all items. Besides, we calculated four composite scores, including scores of the three “higher-order factors” and a total rating across all individual attributes (i.e., total PYD). The scales were proven to possess adequate internal consistency with Cronbach’s alpha values varying between 0.86 and 0.96 and mean inter-item correlations ranging between 0.33 and 0.63 (see Table 1).

### 2.3. Data analysis

In line with previous practices (Zhu and Shek, 2021b; Zhu et al., 2022), we used SPSS Version 26.0 (IBM Corp., Somers, NY, United States) to test and capture changes in students’ “service leadership attributes” as well as “well-being” that occurred from the pretest to the posttest through repeated-measures multivariate general linear model (R-GLM) analyses. For “service leadership attributes” and PYD attributes that involved multiple indicators, a Bonferroni correction procedure was utilized to control the inflated type I error rate due to the multiple numbers of individual tests in detecting the multivariate effect. If the omnibus time effect was significant, we would perform follow-up univariate analyses to further test students’ changes in individual measures. Given that the variables of age, gender, and academic year as between-participant variables in R-GLM did not show significant interactions with the time effect (*F* ranged between 0.19 and 2.51, *ps* > 0.05), final analyses were based on the combined whole sample (*N* = 198; Table 2 shows the correlations among variables).

**Table 2:** Correlations between variables.

Variables	1	2	3	4	5	6	7	8	9	10	11	12	13	14	15	16	17
1. Age	--																
2. Gender^a^	−0.10	--															
3. SLK_pretest	−0.06	0.30^***^	--														
4. SLA_pretest	0.08	0.16^*^	0.32^***^	--													
5. SLB_pretest	−0.03	−0.02	−0.05	0.60^***^	--												
6. LS_pretest	−0.10	−0.02	−0.10	0.26^***^	0.55^***^	--											
7. CBC_pretest	0.02	−0.05	−0.01	0.45^***^	0.79^***^	0.57^***^	--										
8. PI_pretest	−0.01	−0.10	−0.09	0.34^***^	0.77^***^	0.66^***^	0.78^***^	--									
9. GPYD_pretest	0.03	0.02	0.07	0.52^***^	0.81^***^	0.64^***^	0.79^***^	0.76^***^	--								
10. TPYD_pretest	0.02	−0.03	0.00	0.49^***^	0.86^***^	0.67^***^	0.92^***^	0.89^***^	0.95^***^	--							
11. SLK_posttest	−0.04	0.29^***^	0.67^***^	0.33^***^	−0.06	−0.05	−0.08	−0.11	0.04	−0.04	--						
12. SLA_posttest	−0.07	0.14^*^	0.27^***^	0.55^***^	0.29^***^	0.12	0.11	0.07	0.21^**^	0.16^*^	0.35^***^	--					
13. SLB_posttest	−0.01	0.03	−0.02	0.38^***^	0.53^***^	0.37^***^	0.36^***^	0.36^***^	0.45^***^	0.44^***^	0.08	0.72^***^	--				
14. LS_posttest	−0.14^*^	−0.02	−0.10	0.14^*^	0.38^***^	0.57^***^	0.24^***^	0.34^***^	0.39^***^	0.36^***^	−0.13	0.42^***^	0.68^***^	--			
15. CBC_posttest	−0.05	0.00	−0.05	0.25^***^	0.44^***^	0.31^***^	0.34^***^	0.36^***^	0.38^***^	0.40^***^	0.00	0.65^***^	0.82^***^	0.65^***^	--		
16. PI_posttest	−0.02	−0.06	−0.09	0.22^**^	0.50^***^	0.46^***^	0.39^***^	0.52^***^	0.46^***^	0.50^***^	−0.04	0.52^***^	0.78^***^	0.77^***^	0.83^***^	--	
17. GPYD_posttest	−0.05	0.05	0.08	0.35^***^	0.51^***^	0.43^***^	0.38^***^	0.38^***^	0.55^***^	0.50^***^	0.13	0.67^***^	0.84^***^	0.69^***^	0.83^***^	0.78^***^	--
18. TPYD_posttest	−0.05	0.01	0.004	0.31^***^	0.52^***^	0.43^***^	0.40^***^	0.43^***^	0.51^***^	0.50^***^	0.05	0.67^***^	0.87^***^	0.74^***^	0.94^***^	0.90^***^	0.96^***^

^a^1 = male; 2 = female. SLK, service leadership knowledge; SLA, service leadership attitude; SLB, service leadership behavior; LS, life satisfaction; CBC, cognitive-behavioral competence; PI, positive identity; GPYD, general positive youth development qualities; TPYD, total positive youth development score; and ^*^p < 0.05; ^**^p < 0.01; ^***^p < 0.001.

**Figure 1:**
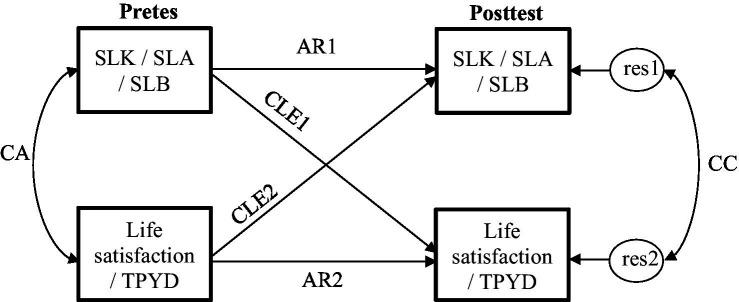
Cross-lagged path analyses of the relationships between service leadership attributes and well-being indicators. SLK, service leadership knowledge; SLA, service leadership attitude; SLB, service leadership behavior; TPYD, total positive youth development score; res, residual; CA, concurrent association; AR1, autoregression of service leadership attributes; AR2, autoregression of well-being indicator; CLE1, cross-lagged effect of service leadership attributes on well-being indicator; CLE2, cross-lagged effect of well-being indicator on service leadership attributes; and CC, correlated change.

The relationship between “service leadership attributes” and “well-being” was examined in six cross-lagged path models using AMOS Version 26.0 (IBM Corp., Somers, NY, United States), with each model involving one “service leadership attribute” (i.e., “knowledge,” “attitude,” or “behavior”) and one “well-being” measure (i.e., “life satisfaction” or TPYD). As shown in Figure 1, after statistically controlling for the concurrent association (CA) between the pretest scores, autoregressions of “service leadership attributes” (AR1), and “well-being” measure (AR2), and the correlated change (CC) indexed by the correlation between the two residuals at posttest, the two cross-lagged effects (CLE1 and CLE2) would estimate the possible overtime effects between “service leadership attributes” and “well-being.” This analytical procedure has also been adopted in previous research (Lin and Shek, 2019; Zhu et al., 2022).

## 3. Results

As shown in Table 1, the R-GLM yielded significant omnibus time effects regarding “service leadership attributes” (*F* = 8.54, *p* < 0.001, *η^2^_p_* = 0.12), PYD attributes (*F* = 5.91, *p* < 0.001, *η^2^_p_* = 0.11), and life satisfaction (*F* = 20.58, *p* < 0.001, *η^2^_p_* = 0.10). Following univariate analyses demonstrated significant improvement in each measure of “service leadership attributes” (*F* = 5.33–20.17, *ps* < 0.05, *η^2^_p_* = 0.03–0.09) and PYD attributes (*F* = 6.13–16.65, *ps* < 0.01, *η^2^_p_* = 0.04–0.09). In general, the students demonstrated significant changes in both “service leadership attributes” and subjective “well-being” from the pretest to the posttest in a positive way.

For the cross-lagged path analyses, all six models were saturated models characterized by zero degrees of freedom and perfect model fitness since all variables in each model were revealed to be interrelated. The results of the cross-lagged path analyses are presented in Table 3. After other types of effects were statistically controlled, four cross-lagged effects were significant. Specifically, while service leadership knowledge and attitude did not have significant cross-lagged effects on life satisfaction, service leadership behavior, and life satisfaction showed significant reciprocal impacts on each other over time (service leadership behavior→life satisfaction: *β* = 0.14, *p* < 0.05; life satisfaction→service leadership behavior: *β* = 0.14, *p* < 0.05). In addition, while there were no significant cross-lagged effects between service leadership knowledge and the total PYD score, both service leadership attitude (*β* = 0.14, *p* < 0.05) and behavior (*β* = 0.36, *p* < 0.01) demonstrated a significant one-way effect on the total PYD score over time. The findings suggest service leadership behavior, in comparison to service leadership knowledge and attitude, was a stronger predictor of later well-being measures.

**Table 3:** Standardized path coefficients of cross-lagged path analyses (*N* = 198).

Models	CC	CA	AR1	AR2	CLE1	CLE2
Service leadership knowledge and life satisfaction	−0.12	−0.10	0.68^***^	0.59^***^	−0.03	0.03
Service leadership attitude and life satisfaction	0.49^***^	0.26^***^	0.58^***^	0.59^***^	0.04	0.02
Service leadership behavior and life satisfaction	0.63^***^	0.55^***^	0.49^***^	0.53^***^	0.14^*^	0.14^*^
Service leadership knowledge and positive youth development	0.09	0.01	0.68^***^	0.52^***^	0.02	−0.02
Service leadership attitude and positive youth development	0.74^***^	0.49^***^	0.61^***^	0.47^***^	0.14^*^	−0.07
Service leadership behavior and positive youth development	0.83^***^	0.86^***^	0.56^***^	0.23^*^	0.36^**^	0.00

CC, correlated change; CA, concurrent association; AR1, autoregression of service leadership attributes; AR2, autoregression of well-being measure; CLE1, cross-lagged effect of service leadership attributes on well-being measure; and CLE2, cross-lagged effect of well-being measure on service leadership attributes; ^*^p < 0.05, ^**^p < 0.01, ^***^p < 0.001.

## 4. Discussion

With regard to the above-mentioned research gaps, there are several constructive responses in this paper. First, we utilized the “Service Leadership Theory” to examine the linkages between leadership attributes and well-being. Second, based on the belief that everyone is a leader and the notion of self-leadership, we tested the ties between “service leadership attributes” and subjective and psychological “well-being” from an intrapersonal perspective. Third, the present study provides an expansion of the limited body of scientific literature regarding leadership specifically situated in Chinese settings. Fourth, we examined the linkage between leadership attributes and well-being in university students (i.e., pre-work context).

With reference to Research Question 1, results indicated that upon completion of the “Service Leadership” course, students showed positive changes. This finding generally supports prior research suggesting that service leadership education is efficacious in fostering leadership attributes along with the well-being of college students (Lin and Shek, 2019; Zhu et al., 2021; Zhu and Shek, 2021b). The present finding is important from the perspective of replication because there are few related studies in the field, especially concerning the online delivery mode. Together with previous findings, the present findings suggest that service leadership education, regardless of its delivery mode (face-to-face or online), is a promising approach to facilitating the well-being of university students. There are studies showing that mental health issues are growing in the higher education sector (Li X. et al., 2021), particularly during the pandemic (Shek, 2021). To tackle the issue, university administrators and educators are searching for ways to promote the mental health of young people. In conjunction with other studies, it is arguable that service leadership education can be meaningfully implemented as a means of promoting life satisfaction and personal growth among university students.

It is noteworthy that the present service leadership education was effective during the pandemic, when lessons have been delivered in online and hybrid modes intermittently since the outbreak of the pandemic in early 2020. One obvious concern regarding online teaching and learning is its effectiveness. In particular, there are very few studies exploring the efficacy of programs in leadership training during the pandemic, which is seen as a health crisis and educational challenge. Consistent with previous studies, the present study showed that students improved after they had taken the online and hybrid “Service Leadership” course. Actually, such positive findings are consistent with research findings based on an online service-learning course on service leadership (Lin et al., 2022; Zhu et al., 2022; Shek et al., 2022b). In short, the present findings reinforce the conclusion that students change in a positive direction after they have taken the online “service leadership” course.

Given that the “Service Leadership” course has been found to be effective in different delivery modes, including face-to-face, asynchronous, and synchronous online modes (Lin and Shek, 2019; Zhu et al., 2021; Zhu and Shek, 2021b), the meticulously designed course content and engaging and reflective learning pedagogies may be the most essential factors contributing to the success of the course. First, the topics covered in the course, such as self-leadership, interpersonal skills, character strengths, and care, are desired in the service era and highly relevant to the students and may cost students much if they take courses on such topics in the commercial field. In addition, the positive beliefs and strength-based perspective (e.g., every student can become an effective service leader by cultivating and applying leadership attributes) underlying the course design empower the students to have more positive learning experiences. Thus, students are intrinsically motivated to learn the topics in the course, which may promote students’ positive changes. Second, we used experiential learning strategies and activities, such as group discussion, group reflection, role play, and debate which are fun and engaging for the students. These activities promote the engagement and responsibility of the students in their studies. In contrast to the didactic teaching and learning in the traditional Chinese setting, this course is relatively interactive. Third, we emphasize personal reflection in this course which would facilitate knowledge acquisition and the development of service leadership attitudes. Active engagement in collaborative learning and reflective activities can promote learning effectiveness (Roberts, 2008; Kolb, 2014), which may cumulatively promote students’ gains and development in this course. Because of these unique features, this course has won several awards, including Bronze Award (Ethical leadership in 2016) and Gold Award (Nurturing Student Well-Being and Purpose in 2021) in QS Reimagine Education Awards, and the University Grants Committee Teaching Award in 2018.

Regarding the second research question on the linkages between core “service leadership attributes” and “well-being,” concurrent correlation coefficients provided support for Hypothesis 2a. The cross-lagged analyses showed service leadership attitude and behavior at the pretest predicted posttest PYD attributes, partially supporting Hypothesis 2b. Meanwhile, leadership behavior and life satisfaction showed bidirectional effects on each other. Overall speaking, the findings showed that leadership attitudes and behavior, but not knowledge, are likely to shape both subjective well-being and psychological well-being over time. The outcome is in line with the prior observation that service leadership knowledge and well-being do not statistically significantly relate to one another (Lin and Shek, 2019). In other words, mere acquisition of service leadership knowledge does not contribute to well-being. This finding is not unexpected because understanding (e.g., the importance of exercise) will not lead to happiness if one does not spend time on exercise. Hence, on top of teaching leadership knowledge, cultivating the service mindset (i.e., attitude) and service practices may be more important in promoting students’ well-being through service leadership education. This may be the reason that service-learning courses, where students develop and apply their leadership qualities through “doing” (i.e., serving needy people), have been found to be especially useful in promoting students’ good feelings about themselves (e.g., Lin et al., 2022; Zhu et al., 2022). Nevertheless, Lin and Shek (2019) did not observe a significant cross-lagged effect of leadership attitude on life satisfaction. To further confirm the findings presented here, replications are needed.

Compared to knowledge, service leadership attitude and behavior exert a stronger influence on the development of mental well-being indicated by PYD attributes. Zhu and Shek (2021b) argued that when students internalize service leadership beliefs (such as the belief that “leadership can be nurtured”) and practice service leadership behavior (such as showing empathy to others), they are more likely to unleash their potential and gain greater personal growth. The present finding also supports the social change model in leadership (Stephens and Beatty, 2015), which holds that leadership cultivation and development would enable transformation in many developmental domains such as social connectedness and positive mentality. In terms of student development, researchers (e.g., Komives et al., 2009) have argued that “knowing” and “being” are important factors in shaping well-being among young people. As service leadership attitudes are “humanistic” and “systemic” in nature (Shek et al., 2021), such as the belief that everybody can be a leader and that an effective leader cares for coworkers, nurturing such attributes among students would eventually contribute to their overall wellness.

We also found the predictive effect of life satisfaction on service leadership behavior characterized by demonstrating competence, care and moral character in leading self and others. It is possible that inherently, a bidirectional relationship exists between leadership behavior and life satisfaction (Zhu and Shek, 2021a). While internalization of service leadership beliefs and application of these beliefs in leadership practice may enable students to enjoy a productive and happy life (Hausler et al., 2017; Zhu and Shek, 2021b), feeling good about oneself and satisfaction with life may, in turn, serve as valuable emotional resources to help students cope and behave more effectively and adaptively (Fredrickson, 2001; Park, 2004). While the reciprocal relationship between individual behavior and life satisfaction has drawn much attention in youth development (e.g., Zhu and Shek, 2021a), it has been understudied in the leadership field. Additional studies are desired to further validate the present discoveries.

In general, the present study findings indicate that it is likely to be more important to nurture the attitudinal and behavioral growth of students (i.e., development of attitudes and behavior) rather than merely knowledge learning (Owen, 2012). Instructors engaged in leadership education will find these observations tremendously instructive and illuminating. While much leadership training often prides itself on equipping and empowering leadership knowledge and skills, leadership educators are expected to clearly recognize that it is far from sufficient, as leadership attitude transformation and behavioral applications are of greater importance for students to harvest a well-lived life than knowledge acquisition. Attitude transformation, however, can never be forced (Owen, 2012). Thus providing the insight that leadership programs should promote student initiative and then enhance acquisition under the mentorship of teachers by creating better opportunities and circumstances.

Although the present study replicated the previous findings and underscored the validity of online delivery of service leadership training during the pandemic, there are several limitations of the study. First, we adopted the one-group pretest-posttest design in this study. While this design is widely adopted in the field, the addition of a control group is important. Without a control group, we are not able to establish a causal relationship between the course effect and student changes. In other words, we are not able to completely rule out alternative explanations (e.g., student maturation and effects of other activities during the course period) in addition to the course effect for the changes in students. However, as the course was offered in 7 weeks during the summer term, the chance for students to naturally have significant improvements in leadership qualities and well-being in such a short time period was not high. In particular, previous studies concluded that university students did not necessarily make gains in their leadership competence and well-being merely because of maturation during the university period (Roohr et al., 2017; Yu et al., 2019). Therefore, the positive changes in service leadership qualities and well-being among students revealed in the present study are unlikely to be entirely attributed to maturation. As for the effects of other activities, as we have not collected such information, it is logically possible that such an alternative explanation exists. However, given the tight time schedule and assessment requirements (students need to do a group project and an individual writing project), students were unlikely to take other courses or programs during the same time period. In short, the present findings provide support for the hypothesis that students changed in a positive direction after taking the course, although we should also note the existence of alternative explanations as constrained by the research design. Nevertheless, future research will benefit from conducting quasi-experimental studies by involving a comparative control group of students who do not join the “Service Leadership” course, which would be methodologically preferred to establish the causal relationship between course effect and student changes.

Second, we only recruited students from one university in Hong Kong, which may limit the generalizability of the present findings to other samples of students. As the “Service Leadership” course adopts experiential and interactive teaching and learning approaches, it is unknown whether students in other universities in and outside Hong Kong also engage in such a learning pedagogy and benefit from their service leadership course participation experience. Hence, future research needs to collect data from more universities within and outside Hong Kong and replicate the present study by involving a more representative sample of university students, which helps generate more conclusive findings that can also be generalized to a broader population.

Third, as the study was conducted in a “pre-work” context, treating university students as leaders and followers, it would be stimulating if data could be collected from the “work” context with the involvement of leaders and followers in the interpersonal context. Thus, another future research direction is to offer service leadership training in the “work” context and investigate the training effect and the associations between service leadership attributes and well-being among both leaders and followers. In particular, such studies can further assess actual leadership behavior in the work context by using different methods, such as observations, diaries, and behavioral checklists.

Fourth, it would be theoretically exciting to study how service leadership attributes of the leaders would influence the psychological well-being of the leaders which would eventually shape the well-being of the followers. This would generate theoretical models on the intimate relationship between leadership and well-being in the leaders themselves and the followers. Finally, as there are three elements of effective service leadership (i.e., competence, character, and care), it would be helpful to examine how different aspects are differentially related to well-being outcomes. This can help researchers to further understand how positive leadership contribute to personal and organizational outcomes, which is relatively neglected in the existing scientific literature on leadership (Hoch et al., 2018).

Despite these limitations, the present study suggests that online “Service Leadership” course during the pandemic is proven to be efficacious in boosting no matter the “service leadership attributes” or “well-being” of university students. Besides, there is evidence supporting the predictive role of both attitudes and behaviors of service leadership on well-being.

## Data availability statement

The raw data supporting the conclusions of this article will be made available by the authors, without undue reservation.

## Ethics statement

The studies involving human participants were reviewed and approved by the Institutional Review Board (or its Delegate) at The Hong Kong Polytechnic University. The patients/participants provided their written informed consent to participate in this study.

## Author contributions

DS designed the research project and contributed to all the steps of the work. XZ contributed to the statistical analyses and drafting work. DD planned the data collection, collected the data, and prepared the data set. LT contributed to literature review and drafting of the manuscript. All authors contributed to the article and approved the submitted version.

## Funding

This work is financially supported by Li and Fung Endowed Professorship in Service Leadership Education, Wofoo Foundation and the Research Matching Fund of the Research Grants Council (R.54.CC.83Y7).

## Conflict of interest

The authors declare that the research was conducted in the absence of any commercial or financial relationships that could be construed as a potential conflict of interest.

## Publisher’s note

All claims expressed in this article are solely those of the authors and do not necessarily represent those of their affiliated organizations, or those of the publisher, the editors and the reviewers. Any product that may be evaluated in this article, or claim that may be made by its manufacturer, is not guaranteed or endorsed by the publisher.
